# Surfactant protein D and bronchopulmonary dysplasia: a new way to approach an old problem

**DOI:** 10.1186/s12931-021-01738-4

**Published:** 2021-05-08

**Authors:** Raquel Arroyo, Paul S. Kingma

**Affiliations:** 1 Division of Neonatology and Pulmonary Biology, Cincinnati Children’s Hospital Medical Center, 3333 Burnet Ave. ML7029, Cincinnati, OH 45229-3039 USA; 2 Airway Therapeutics Inc, Cincinnati, OH 45249 USA; 3 Department of Pediatrics, University of Cincinnati College of Medicine, Cincinnati, OH 45229 USA

**Keywords:** Bronchopulmonary dysplasia (BPD), Surfactant Protein D (SP-D), Recombinant human SP-D (rhSP-D), Alveolar arrest, Lung inflammation

## Abstract

Surfactant protein D (SP-D) is a collectin protein synthesized by alveolar type II cells in the lungs. SP-D participates in the innate immune defense of the lungs by helping to clear infectious pathogens and modulating the immune response. SP-D has shown an anti-inflammatory role by down-regulating the release of pro-inflammatory mediators in different signaling pathways such as the TLR4, decreasing the recruitment of inflammatory cells to the lung, and modulating the oxidative metabolism in the lungs. Recombinant human SP-D (rhSP-D) has been successfully produced mimicking the structure and functions of native SP-D. Several in vitro and in vivo experiments using different animal models have shown that treatment with rhSP-D reduces the lung inflammation originated by different insults, and that rhSP-D could be a potential treatment for bronchopulmonary dysplasia (BPD), a rare disease for which there is no effective therapy up to date. BPD is a complex disease in preterm infants whose incidence increases with decreasing gestational age at birth. Lung inflammation, which is caused by different prenatal and postnatal factors like infections, lung hyperoxia and mechanical ventilation, among others, is the key player in BPD. Exacerbated inflammation causes lung tissue injury that results in a deficient gas exchange in the lungs of preterm infants and frequently leads to long-term chronic lung dysfunction during childhood and adulthood. In addition, low SP-D levels and activity in the first days of life in preterm infants have been correlated with a worse pulmonary outcome in BPD. Thus, SP-D mediated functions in the innate immune response could be critical aspects of the pathogenesis in BPD and SP-D could inhibit lung tissue injury in this preterm population. Therefore, administration of rhSP-D has been proposed as promising therapy that could prevent BPD.

## Bronchopulmonary dysplasia (BPD)

### Incidence and definition

Bronchopulmonary dysplasia (BPD) was first described in 1967 [1] and still remains as the most common complication of preterm birth [2]. Improvements in the clinical management of preterm babies at risk of BPD and the continuous advances in modern medicine keep decreasing the gestational age (GA) for survival of premature infants. Due to increases in survival coupled with the lack of effective therapies for BPD, the incidence of BPD has not decreased but increased lately. The incidence of BPD is influenced by the hospitals, countries – how they manage preterm babies- and also by the diagnosis criteria applied to define BPD [2, 3].

The definition of BPD has evolved since it was first described in 1967. Progress in the understanding of BPD pathophysiology and changes in the clinical management of preterm infants with the tools available in modern medicine, specially related to surfactant therapy, ventilation, CPAP and oxygen support, have pushed the evolution of the BPD definition. The latest definition proposed by Jensen et al. is a modification of the NICHD workshop definition [4] to introduce a severity scale that is based on the level of respiratory support at 36 postmenstrual age (PMA) weeks instead of supplemental oxygen [5]. It classifies BPD severity of infants at 36 PMA weeks as no BPD (no support), grade 1 (nasal cannula ≤ 2 L/min), grade 2 (nasal cannula > 2 L/min or non-invasive positive airway pressure) and grade 3 (invasive mechanical ventilation) [5].

### Pathogenesis

The mechanisms and pathophysiology underlying BPD are complex and not completely understood. BPD affects the epithelial and endothelial components of the lung. Alveolar and vascular development are closely intertwined in the developing fetal lung. Therefore, the epithelial lung damage observed in BPD is accompanied by an impairment of the pulmonary vascular development and angiogenesis in BPD patients [2]. BPD also impacts organs outside the lung such as the brain [2, 6]. In this review we focus on the events or factors that affect the lung epithelia and lead to BPD development. Preterm infants are born with their lungs at early morphogenesis stages, canicular or saccular, instead of the alveolar stage observed in term infants [2, 7, 8]. The intrinsic prematurity of the pre-term lung with low pulmonary surfactant lipid levels, low surfactant protein levels, underdeveloped antioxidant mechanisms, low compliance and inadequate fluid clearance, combined with the presence of inflammation, which is developed as a consequence of several pre- and postnatal factors, causes a heterogenous injury in the lungs that results in BPD. Lungs of BPD patients exhibit fewer secondary septa and alveoli that is translated in disrupted alveolarization, cystic emphysema, fibrosis characterized as fibrotic tissue with widening and thickening of the interstitial spaces, activated pro-inflammatory responses with granulocyte recruitment and increased white blood cells, cytokines, oxidants [2, 4, 9]. All these lung alterations result in the abnormal and inefficient gas exchange and impaired lung mechanics that is observed in BPD patients.

Prenatal factors that increase the risk of BPD development are genetic susceptibility, intrauterine growth restriction (IUGR) that limits lung growth and maturation, maternal smoking, pregnancy-related hypertensive risks and chorioamnionitis [2, 10]. Chorioamnionitis is particularly relevant in the context of this review because it promotes a pro-inflammatory response in the fetal lung. Chorioamnionitis produces inflammation of intrauterine fetal membranes, maternal decidua and amniotic fluid, which most often associated with the colonization by vaginal commensals pathogens such as *Ureaplasma sp.* The fetal lung is exposed to the inflammatory mediators in the amniotic fluid via fetal breathing and resulting in lung inflammation that contributes to BPD development [11, 12]. Animal models of chorioamnionitis have shown that it may also affect the endogenous surfactant protein levels of the fetal lung, including SP-D. The expression of SP-D in the fetal lung was reduced in a mouse model of chorioamnionitis that induced preterm birth 8 h after intraperitoneal lipopolysaccharide (LPS) administration [13]. In contrast, a lamb study found increased SP-D expression in lungs 14 days after intra-amniotic LPS administration where delivery was performed by cesarean [14].

**Fig. 1:**
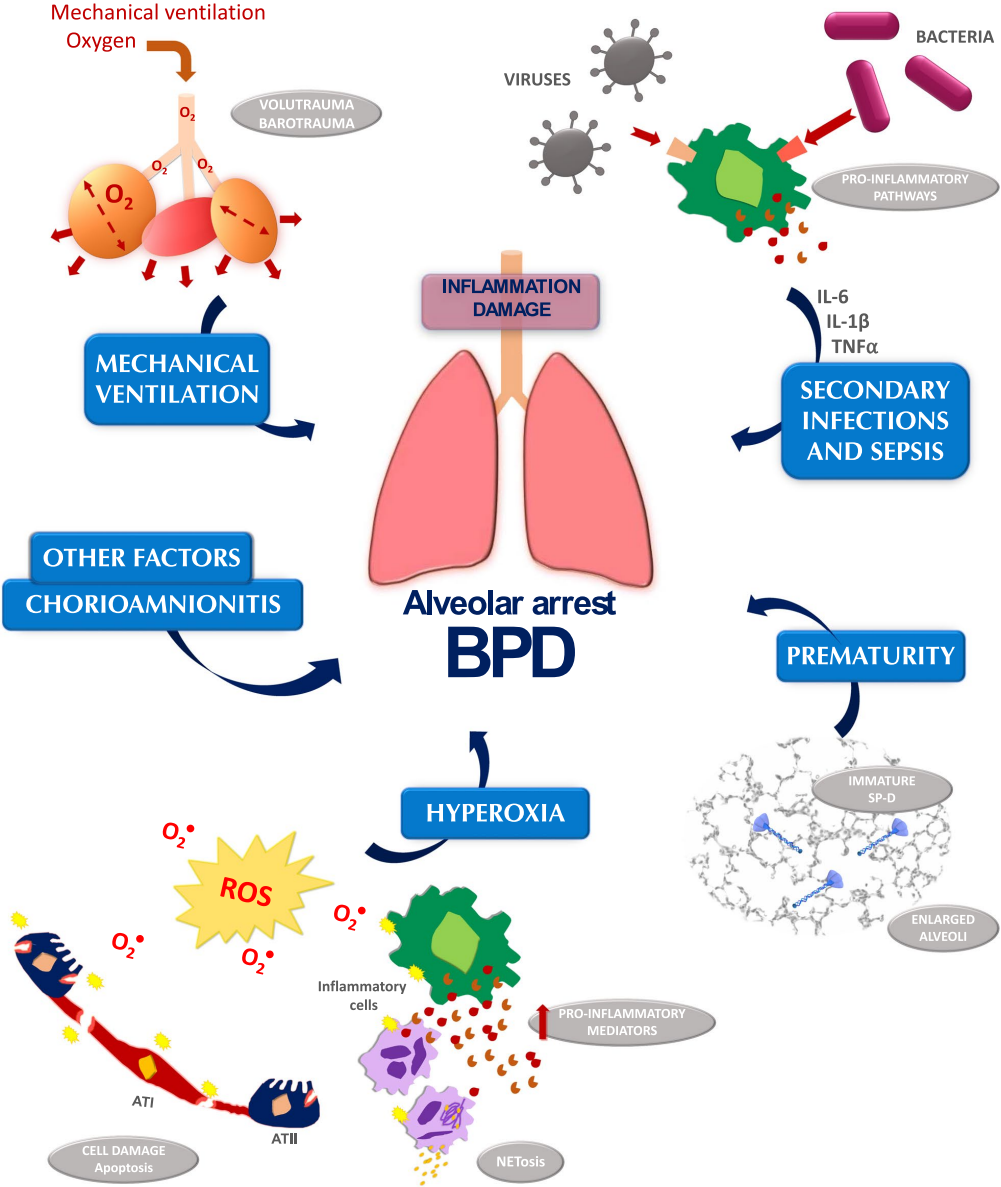
Factors that contribute to alveolar arrest in BPD. Several factors contribute to the development of inflammation in BPD. Mechanical ventilation and oxygen therapy may lead to volutrauma and barotrauma. Hyperoxia induces cell damage, apoptosis and NETosis, which are translated to a pro-inflammatory response with increased production of pro-inflammatory mediators (such as metalloproteinases, cytokines, etc.). Secondary infections and sepsis caused by pathogens (such as viruses and bacteria) activate pro-inflammatory pathways to eliminate the threat, but this inflammatory response promotes the release of pro-inflammatory cytokines (IL-6, IL-1β, TNFα, etc.) which contribute to lung injury. Chorioamnionitis and other factors may also contribute to the development of BPD. The intrinsic prematurity of the lungs from premature infants with enlarged alveolar spaces, typical of canicular or saccular morphological stages, and the immature SP-D at decreased levels also contribute to the development of BPD

Postnatal factors that contribute to BPD are oxygen therapy, mechanical ventilation, postnatal infections -sepsis-, respiratory microbial dysbiosis and vascular events such as patent ductus arteriosus (PDA). Oxygen exposure induces the formation of reactive oxygen species (ROS), which combined with the immature anti-oxidant mechanisms of the premature lung may lead to accumulation of toxic levels of ROS in the alveoli. ROS can damage cell constituents such as membrane lipids and structural proteins leading to cell death and lung tissue damage [10, 15]. During this process, cytokines and chemo-attractant factors are released to recruit more inflammatory cells to the alveoli that also coincides with the activation of the NLRP3 inflammasome. Macrophages and activated neutrophils release even more pro-inflammatory cytokines (e.g. IL-6, IL-1β, IL-8, TNF-α), macrophage inflammatory proteins, metalloproteinases, proteases, neutrophil extracellular traps (NETs). At the same time the production of anti-inflammatory cytokines (e.g. IL-10) is decreased, thus exacerbating inflammation and enhancing the epithelial damage [10, 16–22] (Fig. 1).

Supplemental oxygen can be administered with different techniques that can be non-invasive or invasive in different degrees. In some cases, mechanical ventilation is needed in addition to supplemental oxygen for proper gas exchange and breathing. Besides the oxidative stress induced by the supplemental oxygen administered by mechanical ventilation, mechanical ventilation contributes to BPD by itself (Fig. 1). Although ventilation strategies of preterm babies have improved over the years and techniques have tried to minimize lung injury whenever possible; however, mechanical ventilated lungs are still exposed to artificial high pressures, stretch, high tidal volumes and overextension which increases lung tissue injury and activation of pro-inflammatory cascades, contributing also to BPD [10, 20, 23, 24].

Sepsis is another postnatal factor that also contributes to BPD. Infection by pathogens including gram positive bacteria, gram negative bacteria and fungus boosts an inflammatory response to fight the invading microorganisms that promotes recruitment of inflammatory cells to the lungs and release of pro-inflammatory mediators that cause alveolar damage [25–28] (Fig. 1).

The heterogeneous injury originated in BPD lungs and its progression have been observed by several imaging techniques like magnetic resonance imaging (MRI) and computerized tomography (CT). Ultrashort echo time MRI (UTE-MRI) is a sensitive, nonionizing technique that has demonstrated to reliably identify BPD severity and predict short-term outcomes of patients. UTE-MRI can also distinguish cystic versus normal lung regions, showing that cystic areas are ventilated areas with a higher tidal volume than normal lung areas [29–31]. These novel lung imaging techniques have allowed clinicians and researchers to improve their understanding of the pathology, progression and function of the BPD lung in newborn infants and through childhood.

There are long-term consequences derived from the damage to the lungs of preterm babies during BPD. These patients have impaired post-natal alveolar growth with an abnormal structure and lung function that is clinically manifested in multiple ways. The risk of hospitalization due to complicated respiratory episodes triggered by common viral infections such as Respiratory Syncytial Virus (RSV) or influenza virus is highly increased in this population. During childhood and adulthood, obstructive lung disease with chronic lung dysfunction is observed and they have an increased risk of chronic obstructive pulmonary disease [2]. Therefore, the lung damage induced by BPD is translated to long-term respiratory outcomes that significantly decrease the quality of life of these patients.

### BPD therapies

To date, there are no effective therapies that specifically target the multifactorial complex scenario observed in BPD patients. Strategies regarding oxygen therapy and mechanical ventilation have been improved over the years, decreasing oxygen saturation levels and applying less invasive and gentler ventilation strategies, to reduce the lung injury associated with both measures. In addition, new, less invasive techniques have emerged to administer exogenous pulmonary surfactant at birth that do not require intratracheal intubation. Exogenous pulmonary surfactant has drastically improved survival of preterm infants by facilitating the transition to air breathing of premature infants [32–35]. Despite surfactant therapy, many infants, especially the very preterm ones, still require oxygen supplementation and mechanical ventilation after surfactant therapy to facilitate appropriate gas exchange in the lungs and survive. Despite the promise of surfactant therapy during its early implementation, it has been shown that surfactant therapy itself does not decrease the rate of BPD [35, 36].

There are three therapies that have shown some modest benefits in BPD cohorts that have been reviewed in detail by other authors [2, 35, 37]. First, steroid therapy, as anti-inflammatory drugs, has been shown to reduce inflammation and improve pulmonary function in premature infants, however, corticosteroids bring associated secondary adverse effects that in some cases may outweigh the benefits. Lately, different strategies are being tested to decrease the associated risks and enhance the benefits [2, 37], these strategies include administration of lower doses [38, 39], use of different routes of administration rather than systemic, and co-administration – of budesonide in this case- with exogenous pulmonary surfactant [40, 41]. Second, caffeine therapy has demonstrated that it can reduce the risk of BPD and the duration of mechanical ventilation when administered early in life (before 3 days of life). However, it has a tight therapeutic range and adverse effects such as a pro-inflammatory response have been observed when its levels are out of the appropriate range [42, 43]. Lastly, supplementation with vitamin A is associated with a small reduction in BPD or death at 36 weeks GA, however, this reduction was only observed in children weighing less than 1,000 g [44, 45].

There are new therapies under investigation at preclinical or early clinical stages that could be promising to reduce BPD: stem cell therapy, insulin like growth factor 1 (IGF-1) [35, 37, 46], and recombinant human surfactant protein D (rhSP-D) [47].

## Surfactant protein D (SP-D)

### SP-D

Surfactant protein D (SP-D) is a collectin protein that is found in the lungs as well as in other extra-pulmonary mucosa [48, 49]. In the fetal lung, SP-D has been detected at 10–20 weeks of age and the levels of pulmonary SP-D increase during gestation until term labor [48, 50]. SP-D accomplishes a critical role in the innate immune function of the lungs where it recognizes, opsonizes and promotes the phagocytosis and clearance of invading infectious pathogens [51] and other noxious entities such as nano- or micro-particles accidentally inhaled from the environment [52, 53]. In addition, SP-D regulates inflammatory responses by modulating several inflammatory signaling pathways, such as the toll-like receptor 4 (TLR4) pathway [54, 55]. SP-D also influences pulmonary surfactant lipids homeostasis and ultrastructure [56, 57] with a relevant role in the newborn period by decreasing surfactant pool sizes [58].

**Fig. 2:**
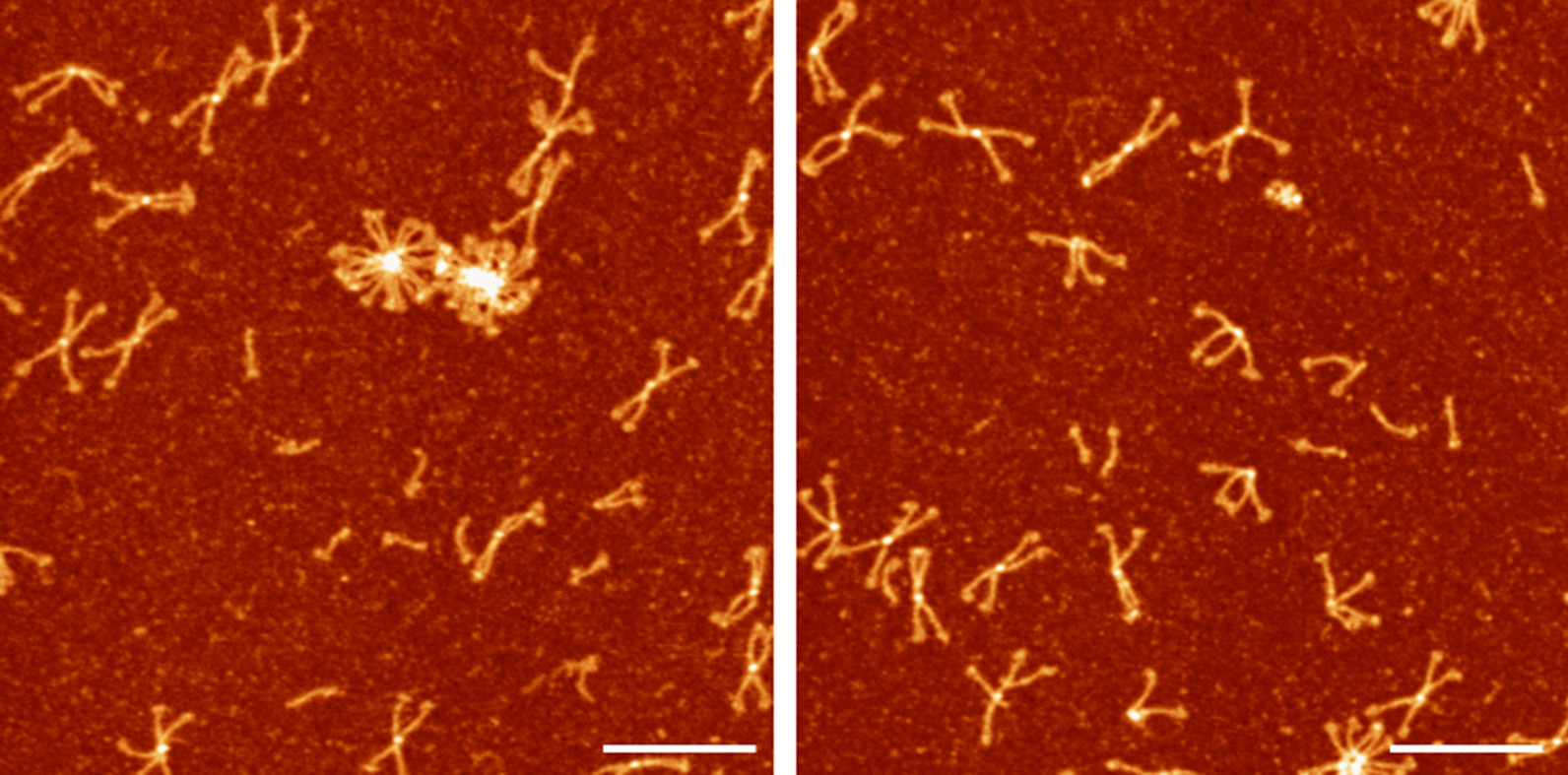
Recombinant human SP-D observed by atomic force microscopy (AFM). AFM images of rhSP-D in which the different oligomeric forms of SP-D can be observed: trimers (single and short stick-like structures), hexamers composed of two trimers observed as V-shaped structures in these images, dodecamers (cross-like structures) the most common specie observed in these images, and higher order oligomers or “fuzzy balls” with a ball-like shape. The scale bar represents 200 nm

SP-D is synthesized by alveolar type II pneumocytes as different oligomeric forms: trimers (minimal protein unit secreted by alveolar type II cells), hexamers, dodecamers and higher order oligomers that are variable in size (also called “fuzzy balls”) [59–61] (Fig. 2). Dodecamers and higher order oligomers have been described to be the most active oligomeric forms of the protein at least for the lectin-mediated functions [62, 63].

Full length recombinant human SP-D (rhSP-D) has been successfully produced in different cell systems with a structure and function comparable to human native SP-D [60–62] raising the possibility of using rhSP-D as a therapeutic agent. Intensive pre-clinical research has been done, including in vitro and in vivo experiments targeting the different aspects of BPD, that demonstrate SP-D could target several factors that participate in BPD pathogenesis. Therefore, in this section we will address the potential of using rhSP-D as a therapy to prevent and/or treat BPD.

### Recombinant hSP-D targets BPD inflammation

**Fig. 3:**
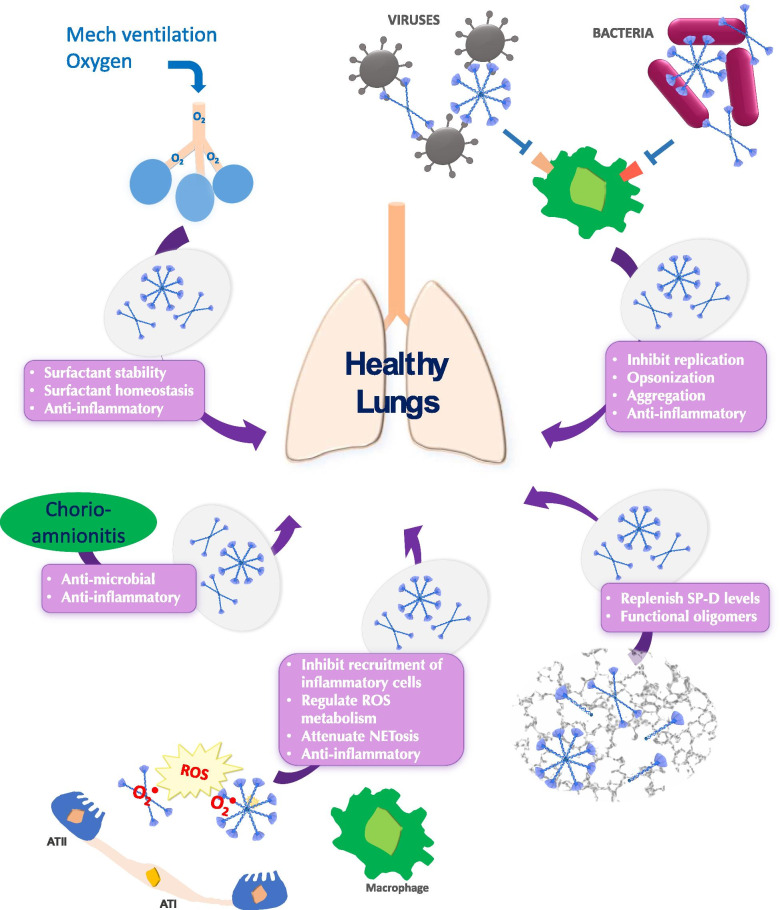
Surfactant protein D (SP-D) mechanisms to counteract BPD inducing factors. SP-D antimicrobial and anti-inflammatory activities could prevent the alveolar damage induced by chorioamnionitis. SP-D contributes to surfactant stability and homeostasis, actions that in combination with its anti-inflammatory action could help to prevent the damage caused by mechanical ventilation and oxygen therapy. SP-D inhibits viral replication, opsonizes and induces aggregation of invading pathogens in secondary lung infections and sepsis to boost their clearance, which is also translated in decreased inflammation. Administration of exogenous SP-D could replenish lung SP-D deficient levels in the preterm lungs and provide oligomerized active SP-D. Last, SP-D could protect the lung from the effects of hyperoxia by inhibiting the recruitment of inflammatory cells, regulating reactive oxygen species (ROS) metabolism, attenuating NETosis, and therefore, decreasing inflammation

Multiple studies have demonstrated that inflammation generated as a result of all the different factors previously described is the central player in BPD pathogenesis, leading to lung injury. The application of rhSP-D as a therapy to prevent and treat BPD would reduce inflammation by down-regulating pro-inflammatory signaling pathways and mediators, and at the same time, it would specifically target some of the external factors, such as neonatal infection, that induce the pro-inflammatory responses (Fig. 3).

Inflammatory pathways mediated by TLR2 and TLR4 are activated in BPD by damage-associated molecular patterns (DAMPs) that are endogenous molecules released by damaged cells, or by pathogen-associated molecular patterns (PAMPs) when there is a pathogen-mediated infection associated [22, 24]. The carbohydrate recognition domain (CRD) of SP-D recognizes and binds to different receptor molecules expressed on the surface of inflammatory cells such as TLR2 [64], the myeloid differentiation factor 2 (MD-2) protein in the TLR4/MD-2 complex [65, 66] and the carbohydrate moiety of CD14 in the CD14/TLR4 complex [67], which inhibits the activation of the NF-κB signaling pathway implicated on amplifying the pro-inflammatory response [54]. DAMPs and PAMPs can also contribute to the development of inflammation in BPD by activation of the NLRP3 inflammasome [18]. It has also been shown that SP-D protects from acute lung injury by suppressing the activation of the NLRP3 inflammasome and the NF-κB pathway [68].

SP-D could also mitigate the inflammatory reactive oxygen species (ROS)-driven response observed in BPD [10, 15]. SP-D binds to the leukocyte-associated immunoglobulin-like receptor 1 (LAIR-1), a receptor in several immunocompetent cells that binds to collagen, inhibiting the production of ROS [69]. Animal models that lack SP-D (*Sftpd*^*−/−*^) have shown increased alveolar concentrations of ROS, confirming a role for SP-D in their modulation [70–72]. ROS can activate the expression of alveolar matrix proteins such as metalloproteinases (e.g. MMP-9), enhancing inflammation in BPD [24], interestingly, SP-D is able to regulate this process, decreasing the production of MMP-2 and MMP-9 by alveolar macrophages [70, 72].

It has been recently published that SP-D attenuates NETosis in human neutrophils [73], decreasing the release of NETs that also contribute to airway inflammation in BPD [19, 21]. Last, it has been shown that SP-D decreases cell apoptosis in-vitro and in-vivo [74–77], and at the same time, favors the elimination of already apoptotic cells by phagocytic cells [78].

In addition to the anti-inflammatory activity, SP-D can target microbial pathogens that colonize the lungs when sepsis and/or secondary infections contribute to inflammatory injury leading to BPD. SP-D recognizes and binds to glycosylated determinants on the surface of bacteria (e.g. lipopolysaccharide in gram negative bacteria [79, 80]), viruses (e.g. F-protein in respiratory syncytial virus (RSV) [81] or the spike-protein of SARS-CoV [82] and SARS-CoV-2 [83]) and fungi (β-glucans and mannose in the wall e.g. in *Candida albicans*) (interactions of SP-D with different pathogens have been reviewed in previous works: [55, 84–88]). This action is mediated by the carbohydrate recognition domain of SP-D and it is calcium-dependent. Binding of pathogens promotes opsonization, aggregation and uptake by phagocytic cells or clearance by the mucociliary escalator, in which higher order oligomers of SP-D (dodecamers and structured multimers or “fuzzy balls”) seem to be more effective in these lectin-mediated functions of SP-D [63, 85, 89–92] (Fig. 3). SP-D-promoted neutralization and removal of pathogens is accompanied by a decreased in the inflammatory response boosted by these microbes, since the insulting agent is being cleared or neutralized avoiding the initiation and exacerbation of the inflammatory pathways. Reduced concentrations of alveolar inflammatory cells and pro-inflammatory cytokines (e.g. IL-6, TNF-α, IL-1β) have been observed in animal models for bacterial and viral respiratory infections and sepsis after treatment with exogenous SP-D, and in animals expressing SP-D versus their *Sftpd*^*−/−*^ counterparts (lacking SP-D) [68, 81, 93–97]. Therefore, a similar effect could be expected in BPD patients if SP-D could promote the clearance of the pathogens that induce secondary infections and sepsis.

In vivo studies that have used different animal models to mimic BPD by exposing preterm animals to hyperoxia and/or mechanical ventilation or sepsis, have shown the important role for SP-D in preventing BPD outcome. Decreased pulmonary levels of collectins have been reported in a model of chronic lung injury induced by mechanical ventilation and oxygen, and in a model of prenatal sepsis in preterm baboons [98, 99]. In a neonatal mouse model that mimicked the hyperoxia exposure in BPD, SP-D showed a protective effect from hyperoxia by modulating the pro-inflammatory response (pro-inflammatory cytokine levels e.g. IL-6, TNF-α were reduced) and antioxidant systems [100]. Last, it has been shown that the addition of rhSP-D to a commercial surfactant inhibited the lung inflammation triggered by ventilation and oxygen administration in premature newborn lambs, while the group that received surfactant alone, without rhSP-D supplementation, showed a significant different increased inflammatory response. The treatment with rhSP-D reduced the total inflammatory cell counts in BALF, the neutrophil elastase activity in tissue and IL-8 concentration in lung [47].

### SP-D in premature infants

The prematurity and underdevelopment of the lungs in preterm infants influence endogenous pulmonary SP-D concentrations and functionality. In ventilated preterm infants that received exogenous pulmonary surfactant treatment during their first week of life, low pulmonary SP-D concentrations at postnatal days 2 and 3 correlated with a worse outcome measured as supplemental oxygen needs at 28 postnatal days. Specifically, infants with low SP-D levels were still on supplemental oxygen therapy at postnatal day 28 while infants with higher SP-D levels were on room air [101]. Lower pulmonary SP-D concentrations at postnatal day 1 have been observed in preterm infants that developed chronic pulmonary disease compared to those that did not [102]. In addition to lower SP-D concentrations, SP-D function was evaluated by binding assays, showing low activity associated with a predominance of low oligomeric forms of SP-D (trimers, which have been associated with lower lectin activity of the protein) in the BALF from preterm infants that developed chronic lung disease [102]. Another study has shown that the premature lung of preterm infants responds to sepsis by increasing SP-D pulmonary levels, an effect that is not observed in the absence of sepsis onset and suggesting that SP-D is a natural tool in the response of the preterm lung to fight infection [103]. All these findings highlight the role that the decreased levels and activity of the SP-D found in the preterm lung play in the development of inflammation, infections and lung injury in this critical population.

The early administration of rhSP-D could replenish deficient endogenous SP-D levels at birth and during the first days of life, providing properly oligomerized and active SP-D (dodecamers and higher order oligomers) to protect the preterm lung from inflammation and lung injury, and also, to provide an adequate defense against infectious pathogens. SP-D also plays a role in the decrease of surfactant pool sizes after birth, converting large aggregates into small aggregates enabling proper surfactant lipids homeostasis [58]. Further research is needed to determine the specific determinants and characteristics of the pulmonary surfactant aggregates that trigger SP-D regulation of surfactant pool sizes, such as the presence of immature surfactant structures or perhaps oxidized or inactivated surfactant aggregates produced by regular breathing and maybe enhanced by inflammation.

In summary, in this section we have reviewed the actions of SP-D that could play a role in preventing BPD. Administration of rhSP-D could prevent the alveolar lung injury observed in BPD patients, mainly caused by an exacerbated inflammatory response originated by multiple factors like intrinsic lung prematurity, chorioamnionitis, postnatal sepsis, mechanical ventilation and oxygen supplementation. The inflammatory response originated by those different factors is mediated by signaling pathways where SP-D has a modulating role, at the same time, rhSP-D could promote the clearance of pathogens and intermediates that stimulate those pathways. Last, rhSP-D could facilitate normal surfactant homeostasis and protect surfactant from inactivation.

### Advantages of rhSP-D therapy over other commercial or experimental drugs

Preterm infants usually require intense hospital care and high medical needs to overcome the difficulties associated with their immature organs, and to ensure proper development. Safety is a high concern when developing and testing new investigational therapies in this preterm population, since they are more fragile than infants and adults. Recombinant human SP-D is a recombinant version of a naturally occurring protein in the human lung that is already present in the lungs of preterm infants at birth, but in a deficient concentration and not in a fully functional form. Since SP-D is a natural occurring protein in the human lung instead of a synthetic artificial drug, the administration of rhSP-D as a therapy to prevent BPD should not bring any significant clinical safety and toxicology concerns.

Finding appropriate delivery techniques is another difficulty that could be encountered to administer a therapy that targets the lungs of the preterm infants. Babies at risk of BPD frequently need exogenous pulmonary surfactant therapy as part of their standard treatment, which is administered by intratracheal instillation with a catheter inserted through the endotracheal tube. This route of delivery requires either intubation of the infant or using less invasive surfactant administration (LISA) techniques that use a less invasive thin and soft catheter [104]. Administration of rhSP-D in different animal models, including one for BPD in preterm lambs, has successfully been performed by intratracheal instillation, obtaining a good clinical outcome and without problems associated to the administration route [47, 95, 96]. Therefore, the same delivery techniques that are already in place to administer exogenous pulmonary surfactant to preterm babies could be potentially use for the delivery of rhSP-D. It has been reported that SP-D has a half-life of 13 h in lungs after intratracheal administration to adult mice [105] and a half-life of 9 h in premature lungs of 10-day old rats with the same administration route [106], therefore, the half-life of the protein would be appropriate to allow for a convenient daily dose regimen for administration. The volume of administration required could be a limiting factor since lungs do not tolerate high volumes of liquid, however, surfactant studies suggest that volumes as high as 4 mL/kg body weight per dose are well tolerated. In addition, volume limitations could be overcome by adjusting the concentration of the clinical presentation of rhSP-D to increase the protein and reduce the volume. The non-toxic and safe profile of intratracheal dosed rhSP-D was recently shown in a premature lung model using 10-day old rats [106]. Repetitive intratracheal administrations of rhSP-D at a dose 8 mg/kg during 14 days were well tolerated by the rats without evidence of an inflammatory response or histological signs of lung toxicity [106]. Besides intratracheal administration, other delivery techniques could be explored. A recent *in-vitro* study has shown that exogenous pulmonary surfactant could facilitate the distribution of rhSP-D. The study demonstrated that pulmonary surfactant is able to transport rhSP-D long distances over air–liquid-like interfaces, which may help rhSP-D to reach to alveoli [107]. It has beenpublished that nanoparticles composed of PLGA (poly lactic acid-co-glycolic acid) were applied as a vehicle to deliver rhSP-D to the lung of mice without exhibiting any associated cell toxicity and providing a sustained rhSP-D released in time that could prevent repetitive doses [108]. Formulations of lyophilized rhSP-D could be developed in the future, maybe enabling the use of nebulizers to administer rhSP-D by nebulization.

### The challenge of manufacturing rhSP-D at commercial scale

A major obstacle in the development of SP-D as a therapeutic over the years has been the complexity of producing and manufacturing a recombinant version of SP-D at clinical scales. As we mention earlier, human native SP-D is naturally assembled as different oligomeric forms: trimers, hexamers, dodecamers and high order oligomers variable in size [61] (Fig. 2). The dodecamer of the protein has a molecular weight of 520 kDa with a cross-like shape in which the length of the enclosed hexamers is ~ 136 nm, therefore, SP-D is a large protein and structurally far from a globular assembly [60]. In addition, when the protein is synthesized by type II pneumocytes, it undergoes post-translational modifications like hydroxylation and glycosylation processes that only mammalian cell systems can accurately mimic when the protein is produced as a recombinant version in the lab [109, 110]. Unfortunately, mammalian cell systems usually produce low recombinant protein yields compared to bioengineering systems that use other organisms like bacteria such as *Escherichia coli,* or fungi such as *Pichia pastoris*. Recombinant SP-D has been produced in different systems, including mammalian cells (CHO-K1, HEK-293), bacteria (*Escherichia coli*) and fungi (*Pichia pastoris*). Currently, mammalian cells are the only system that produced full length, post-translationally modified, and fully oligomerized rhSP-D [111]. Some studies have engineered a truncated version of SP-D (rfhSP-D) of 60 kDa that contains a trimeric neck and CRD, including a short region of the collagen domain (8 triplets) [112, 113]. The main advantage of this truncated version is that protein production using a bacterial system is easier and facilitates high yields, however, the protein only form trimers. While these rfhSP-D trimers still exhibit carbohydrate binding activity and some anti-inflammatory properties of SP-D, they are less active and potent than the dodecamers and higher order oligomers of the full-length protein. Therefore, a higher protein concentration of the rfhSP-D could be needed for adequate therapeutic activity [112, 114–116]. In addition, some SP-D mediated functions require the complex full length protein assembly (dodecamers) such as aggregation of pathogens and regulation of pulmonary surfactant homeostasis, in which the trimeric form has failed to perform these functions [63, 91]. Therefore, the rhSP-D produced has to be characterized with the appropriate quality controls to ensure protein structure, distribution of oligomeric forms, stability and biological activity are maintained during manufacturing, storage and administration, which in the case of rhSP-D with its particular structure and dynamic character is challenging.

Despite these difficulties, there are two biotech companies currently working on producing rhSP-D (Airway Therapeutics Inc., U.S.A.) or the truncated version rfhSP-D (Trimunocor, U.K.) with the aim of using them at clinical trials targeting BPD. Trimunocor is a biotech company based in Hertfordshire (United Kingdom) that is developing and manufacturing the recombinant trimeric fragment of SP-D (rfhSP-D) using *E.coli* as a production system. Trimunocor is in the GMP manufacturing process of CARE-PD as per the company’s website (www.trimunocor.com). Airway Therapeutics Inc., a biotech company based in Cincinnati and Atlanta (U.S.A.) is developing a full length recombinant human SP-D (rhSP-D) in a human cell line GlycoExpress (GEX®) developed by Glycotope GmbH (Germany). This is a human cell line that is capable of producing a full length glycosylated rhSP-D. Airway Therapeutics is planning on using rhSP-D as preventive treatment for BPD with entry into clinical studies in 2021 (as per www.clinicaltrials.gov and per Airway Therapeutics Inc. website: www.airwaytherapeutics.com).

## Conclusions

BPD is a complex and multifactorial syndrome in which lung inflammation is the leading factor that induces lung injury and results in a deficient gas exchange in the lungs. There are no effective therapies that target the multifactorial components in BPD to date and only discrete effects have been shown by corticosteroids, vitamin A and caffeine in reducing some of the BPD outcomes. Recombinant human SP-D (rhSP-D) could be a potential successful biologic therapy for BPD that could target several pathological mechanisms contributing to BPD development. RhSP-D has a high safety profile that mimics a native protein that is normally present in the lung but deficient in preterm infants. The innate immune character of SP-D provides a multitarget response that could act on different factors that contribute to inflammation and injury of the BPD lung, positioning rhSP-D as a multi-target therapy to prevent and treat BPD. Multiple studies have suggested and supported the therapeutic potential of SP-D and recent advances in the manufacturing of this protein may finally lead to the testing of this novel agent in the near future.

## Data Availability

Not applicable.

## Abbreviations

BPD: Bronchopulmonary dysplasia
CRD: Carbohydrate recognition domain
CT: Computerized tomography
DAMPs: Damage-associated molecular patterns
GA: Gestational age, IGF-1: insulin like growth factor 1
IUGR: Intrauterine growth restriction
LISA: Less invasive surfactant administration
LPS: Lipopolysaccharide
NETs: Neutrophil extracellular traps
NICHD: Eunice Kennedy Shriver National Institute of Child Health and Human Development
PAMPs: Pathogen-associated molecular patterns
PDA: (Patent ductus arteriosus
PLGA: Poly lactic acid-co-glycolic acid
PMA: Postmenstrual age
rhSP-D: Recombinant human surfactant protein D
ROS: Reactive oxygen species
SP-D: Surfactant protein D
UTE-MRI: Ultrashort echo time magnetic resonance imagining
